# Regional heritability mapping method helps explain missing heritability of blood lipid traits in isolated populations

**DOI:** 10.1038/hdy.2015.107

**Published:** 2015-12-23

**Authors:** M Shirali, R Pong-Wong, P Navarro, S Knott, C Hayward, V Vitart, I Rudan, H Campbell, N D Hastie, A F Wright, C S Haley

**Affiliations:** 1 MRC Human Genetics Unit, MRC Institute of Genetics and Molecular Medicine, University of Edinburgh, Edinburgh, UK; 2 Roslin Institute and Royal (Dick) School of Veterinary Studies, University of Edinburgh, Midlothian, UK; 3 Institute of Evolutionary Biology, University of Edinburgh, Edinburgh, UK; 4 Croatian Centre for Global Health, Faculty of Medicine, University of Split, Split, Croatia; 5 Centre for Population Health sciences, University of Edinburgh, Edinburgh, UK

## Abstract

Single single-nucleotide polymorphism (SNP) genome-wide association studies (SSGWAS) may fail to identify loci with modest effects on a trait. The recently developed regional heritability mapping (RHM) method can potentially identify such loci. In this study, RHM was compared with the SSGWAS for blood lipid traits (high-density lipoprotein (HDL), low-density lipoprotein (LDL), plasma concentrations of total cholesterol (TC) and triglycerides (TG)). Data comprised 2246 adults from isolated populations genotyped using ∼300 000 SNP arrays. The results were compared with large meta-analyses of these traits for validation. Using RHM, two significant regions affecting HDL on chromosomes 15 and 16 and one affecting LDL on chromosome 19 were identified. These regions covered the most significant SNPs associated with HDL and LDL from the meta-analysis. The chromosome 19 region was identified in our data despite the fact that the most significant SNP in the meta-analysis (or any SNP tagging it) was not genotyped in our SNP array. The SSGWAS identified one SNP associated with HDL on chromosome 16 (the top meta-analysis SNP) and one on chromosome 10 (not reported by RHM or in the meta-analysis and hence possibly a false positive association). The results further confirm that RHM can have better power than SSGWAS in detecting causal regions including regions containing crucial ungenotyped variants. This study suggests that RHM can be a useful tool to explain some of the ‘missing heritability' of complex trait variation.

## Introduction

Blood lipoprotein concentration is an important risk factor for coronary heart disease and stroke (Lewington *et al.*, 2007). The main lipoproteins in blood include high-density lipoprotein (HDL) and low-density lipoprotein (LDL) levels and plasma concentrations of total cholesterol (TC) and triglycerides (TG). These traits have moderate to high heritability: 40–60% for HDL, 40–50% for LDL, 35–48% for TG (Weiss *et al.*, 2006; Kathiresan *et al.*, 2007) and 41–53% for TC (Bielinski *et al.*, 2006; Velásquez-Meléndez *et al.*, 2007). Large meta-analysis (Teslovich *et al.*, 2010; Surakka *et al.*, 2015) of genome-wide association studies (GWASs) in blood lipid traits uncovered associations explaining only a fraction of the heritabilities of these traits. In most GWASs, single single-nucleotide polymorphism (SNP) analysis (SSGWAS) has been used to determine associated variants and subsequently their contribution to complex trait variation. This method has been shown to lack power for the detection of rare genetic variants (Bodmer and Tomlinson, 2010) because of low linkage disequilibrium between rare alleles and genotyped SNPs on the SNP genotyping array (Zeggini *et al.*, 2005). Nagamine *et al.* (2012b) suggested that the regional heritability mapping (RHM) method can uncover genetic variance not identified by SSGWAS. The RHM method uses a relationship matrix between individuals based on SNP information from short regions of genome to estimate the trait variance explained by each region and localize variation. The main objective of this method is to uncover the variance contributed by the combined effect of both common and rare alleles in a region that may be missed by the SSGWAS scan. Uemoto *et al.* (2013) demonstrated the advantage in power of RHM in comparison with SSGWAS and some gene-based association methods, such as versatile gene-based association study (Liu *et al.*, 2010), SNP-set (Sequence) Kernel Association Test (Wu *et al.*, 2011) and canonical correlation analysis (Tang and Ferreira, 2012), using simulations based on real genotype data from a human population and a wide range of scenarios for quantitative trait loci (QTLs) with both common and rare alleles. Uemoto *et al.* (2013) demonstrated that the power of RHM to detect QTL is greater than the other methods studied, with causative variants at both low (0.0005<MAF<0.10) and high (MAF >0.10) minor allele frequencies (MAFs). They reported that the power of these gene-based association methods was strongly affected by the QTL MAF. For example, the power of versatile gene-based association study decreased for the detection of low MAF QTLs, whereas that of SNP-set (Sequence) Kernel Association Test, which was developed as a rare-variant association test (Wu *et al.*, 2011), was reduced for high MAF QTLs (Uemoto *et al.*, 2013). Other region-based analyses that account for familial data structure may be similar to RHM in power. For example, SNP-set (Sequence) Kernel Association Test power can be improved by changing the β-weights (Belonogova *et al.*, 2013), and specific family-based versions of the approach are also available (Chen *et al.*, 2013; Svishcheva *et al.*, 2014).

The aims of this study were to identify regions and SNPs that contribute to variation in blood lipid traits using RHM and SSGWAS analysis in three isolated Croatian populations and to compare the effectiveness of the RHM approach in capturing the heritability of blood lipid traits with SSGWAS in these populations. We compared our results with those of a large meta-analysis for validation.

## Materials and methods

### Populations, phenotypic and genotypic data

A total of 2246 adult volunteers from three Southern European populations in the city of Split and the islands of Vis and Korcula on the Dalmatian coast of Croatia participated in 3 epidemiological field studies. All cohort studies were approved by the Ethical Committee of the Medical School, University of Zagreb and the Multi-Centre Research Ethics Committee for Scotland. All participants gave written informed consent. Fasting plasma levels of four blood lipid traits (LDL, HDL, TC and TG) were recorded. Supplementary Table 1 presents the phenotypic mean, s.d. and sample size for each of four traits in these populations. Before merging the data together, each population was adjusted for the fixed effects, sex, age and age squared by fitting a linear regression model and the residuals were rank transformed to the standard normal distribution, separately. The adjusted and rank-transformed data were used as the phenotypes for both SSGWAS and RHM analysis.

The samples were genotyped using 300K SNP genotyping arrays (Illumina Human Hap300 for Vis and Illumina Inc. (San Diego, CA, USA) CNV370 for Korcula and Split). Quality control procedures were performed per SNP and per individual. SNPs with MAF <0.001, Hardy–Weinberg equilibrium (*P*<10^−8^) and call rate <0.98 were discarded. Individuals were excluded with a call rate of <0.98. After these quality control stages, 2246 individuals remained, and 268 651 autosomal SNPs were common to all the population samples and were used in our analysis. Genotypic quality control was done using the GenABEL library version 1.6-5 (Aulchenko *et al.*, 2007) in R (R Development Core Team, 2011, Vienna, Austria, version 2.12.2).

### Statistical analysis

#### Single SNP genome-wide association method

For all four traits, SSGWAS analysis was performed using the GenABEL package in the R environment. The whole genome kinship matrix was constructed using pair-wise identities by state of all autosomal SNPs in the data set that passed the quality control criteria using the ibs function of GenABEL (option weight=‘freq') as described by Aulchenko *et al.* (2007). The association test was performed fitting the whole genome kinship matrix as a random effect to account for relatedness using the polygenic and mmscore functions of GenABEL (Chen and Abecasis, 2007) in R.

#### RHM analysis method

In the RHM method, the genome was divided into regions using sequential windows of genotyped markers to assess the contribution from each region to the trait variance. A random effects model was applied to the adjusted and rank-transformed phenotypes. For this study two random effects were included: the regional genomic polygenic and whole genome polygenic matrices, as described by Nagamine *et al.* (2012b), using the ASReml software (Gilmour *et al.*, 2002). To evaluate the evidence for a regional heritability >0, for each window, a likelihood ratio test statistic compared the full model including a regional effect with the null model (no regional heritability) that just included the relationship matrix derived using all autosomal SNPs in the data set that passed the quality control criteria. The full model and the null model are as below:


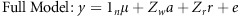



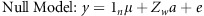


where *y* represents the adjusted and rank-transformed phenotype of interest, *n* is the number of individuals, *μ* is the overall mean, *a* is the whole genome additive genetic effect, matrix *Z*_*w*_ is the whole genome relatedness matrix, *r* is the regional additive genetic effect, matrix *Z*_*r*_ is the regional relatedness matrix and *e* is the vector of residuals. The regional and whole genome genetic values were estimated using a relationship matrix derived from markers as described by Nagamine *et al.* (2012a) by using the formulae below:


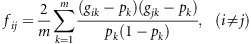



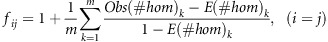


where *f*_*ij*_ is the genomic relationship between individuals *i* and *j*, *g*_*ik*_ (*g*_*jk*_) is the genotype of the individual *i* (*j*) at the *k*-th SNP (coded as 0, 0.5 and 1, for AA, BA and BB, respectively), *p*_*k*_ is the frequency of the allele B of *k*-th SNP and *m* is the number of SNPs used for relationship estimation, representing the total number of autosomal SNPs for the whole genome relationship matrix or the number of SNPs in the region for the regional genomic relationship matrix. *Obs*(#*hom*)_*k*_ and *E*(#*hom*)_*k*_ are the observed number and expected number of homozygous genotypes under Hardy–Weinberg equilibrium at the *k*-th SNP. Whole genomic, regional genomic and residual variances were 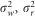 and 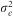, respectively. The variance of the adjusted and rank-transformed phenotype of interest, 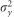, was calculated as 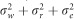 and therefore the whole genome and regional heritabilities were 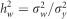 and 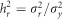, respectively.

In the RHM analyses, the genome was scanned using regions (windows) spanning 100 consecutive genotyped SNPs. Between the sequential windows we allowed for a 50-SNP overlap as explained by Nagamine *et al.* (2012b). Using the relationship matrix obtained from the SNPs within a window enables the contribution of this region to the trait variance to be estimated and regions affecting the trait to be identified. To fine-map associated variants, the 100 top windows based on likelihood ratio test statistic values of 100-SNP window tests were further analyzed using sequential 10-SNP windows with 5-SNP overlap. This potentially allows narrow down the regions of the genome associated with the trait of interest.

#### Significance thresholds

To determine the significance thresholds for SSGWAS and RHM, a Bonferroni correction based on the number of independent tests performed in each scenario was used to obtain a genome-wide 5% significance threshold. In the SSGWAS method, this gave a −log *P*-value threshold of 6.73, as 268 651 autosomal SNPs were used in this analysis. For the RHM method, in 100-SNP windows with 50-SNP overlap and 10-SNP windows with 5-SNP overlap, 5373 and 53 732 windows were tested, respectively, and half that number was used to estimate the Bonferroni corrected significance thresholds and account for overlapping windows (as described by Nagamine *et al.*, 2012b). This resulted in thresholds of 4.73 −log *P*-value for 100-SNP windows with 50-SNP overlap, and 5.73 −log *P*-value for 10-SNP windows with 5-SNP overlap. To balance between type I and type II errors, we considered the suggestive level of significance, defined as the level at which one false positive per genome scan is expected (Rao and Gu, 2001). The genome-wide suggestive significance thresholds obtained using Bonferroni correction were 5.43, 4.43 and 3.43 −log *P*-values for SSGWAS, 10-SNP and 100-SNP windows, respectively.

## Results

### High-density lipoprotein

**Figure 1:**
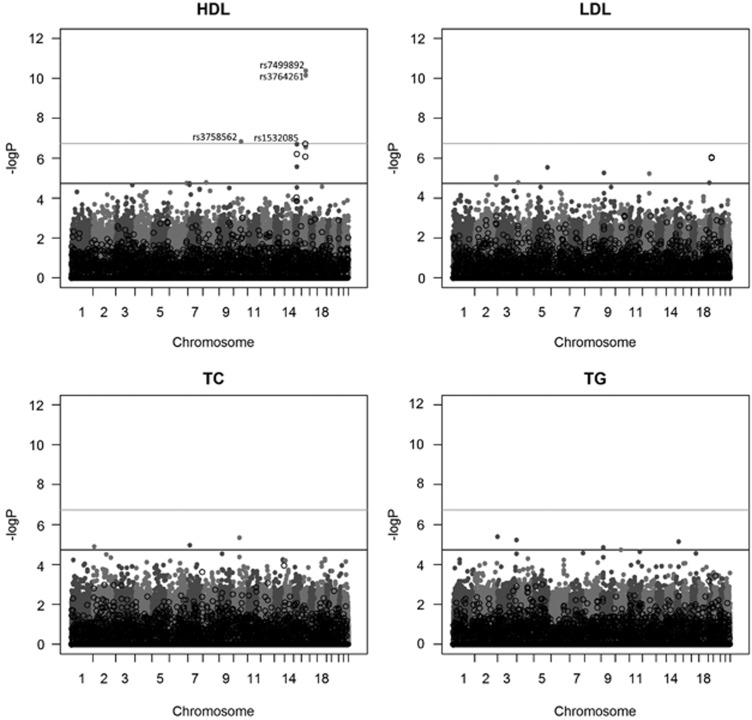
Manhattan plot of results from SSGWAS in gray solid circle and RHM with 100-SNP windows in black filled circle for four blood lipid traits: HDL, LDL, TG and TC. The gray horizontal line indicates the significant threshold level of genome-wide significance at 5% by Bonferroni correction of 6.73 for SSGWAS and the black horizontal line indicates the significant threshold levels of genome-wide significance at 5% by Bonferroni correction of 4.73 for RHM with 100-SNP windows.

Figure 1 shows the Manhattan plot for HDL using the SSGWAS method. Three SNPs associated with HDL were detected by SSGWAS at the 5% significance level after Bonferroni correction. Two of these variants (‘rs7499892' and ‘rs3764261') were close together on chromosome 16 and the third SNP (‘rs3758562') on chromosome 10. Furthermore, a SNP on chromosome 15 (‘rs1532085') was significant at the suggestive level after Bonferroni correction. Supplementary Table 2 presents SSGWAS results for 10 top SNPs with the −log *P-*value of >5.00 for HDL.

Using the RHM method, the estimated heritability of HDL over the whole genome (estimated by averaging the whole genome heritabilities across all 100-SNP windows) was 45.8% (s.e. 6.6%). Two significant windows associated with HDL were detected using the 100-SNP window analysis at the 5% significance level after Bonferroni correction, one on chromosome 15 and the other on chromosome 16 (Table 1). Fine-mapping RHM analysis (using 10-SNP windows) of the associated region on chromosome 15 showed that the region with 3.5% heritability contains the SNP (‘rs1532085') that was suggestive and not genome-wide significant in the SSGWAS analysis. In addition, fine-mapping of the significant region on chromosome 16 indicated that the window contains two significant overlapping 10-SNP windows. The region covering these two overlapping 10-SNP windows each with 4.0% heritability harbors the SNP ‘rs3764261' identified by SSGWAS. Supplementary Table 3 presents RHM results for 10 top 100-SNP windows for all traits of interest.

### Low-density lipoprotein

Using SSGWAS, no SNP associated with LDL was detected at the genome-wide significance level. The estimated heritability of LDL averaged over the RHM genome scan using 100-SNP windows was 47.4% (s.e. 6.5%). RHM identified a region on chromosome 19 from 49 455 904 to 50 999 246 base pair associated with LDL. This region encompassed two genome-wide significant overlapping 100-SNP windows explaining 4.8% and 6.2% of the trait variance for LDL, respectively. The fine-mapping analysis using 10-SNP windows narrowed the region down to a single 10-SNP region with an estimated 7.3% heritability.

### TC and TG

Using the RHM genome scan, the estimated heritability for TC was 42.6% (s.e. 6.4%) and for TG was 18.8% (s.e. 6.3%). However, both the RHM and SSGWAS analyses failed to detect SNPs and/or regions associated with these traits at the genome-wide or suggestive significance levels. Supplementary Table 2 presents SSGWAS results for SNPs with −log *P-*values of >5.00 for TC and TG.

## Discussion

### RHM vs SSGWAS

Table 2 presents the estimated variances of the significant SNPs detected by the SSGWAS method and their regional variance estimated by the RHM method compared with the SNP variances suggested by meta-analysis. On chromosome 10, the significant SNP (‘rs3758562') identified by SSGWAS to be associated with HDL was not confirmed by the RHM method as the region containing this SNP was not significantly associated. This SNP and the region containing it was also not detected in the meta-analysis of Teslovich *et al.* (2010) and in the most current meta-analysis report in HDL by Surakka *et al.* (2015) and also no report was found for any significant SNP around this region in GWAS catalog (http://www.genome.gov/gwastudies) for HDL. This result may suggest that SSGWAS detected a false positive and RHM method may have avoided this problem. Furthermore, on chromosome 15, the suggestive SNP (‘rs1532085') by SSGWAS explained 1.2% of total trait variance. However, the 100-SNP window containing this SNP was found to be highly significant by the RHM method, explaining 2.7% of total trait variance. Comparing these results with the meta-analysis of Teslovich *et al.* (2010) reveals that this SNP is one of the significantly associated variants with HDL with 0.9% heritability based on its reported allele frequency and estimated effect. This result indicates that the RHM method has the power to detect truly associated variants that may not reach the genome-wide significance level and hence may be missed by SSGWAS analysis. Finally, on chromosome 16, the SSGWAS analysis identified one significantly associated variant for HDL, ‘rs3764261', explaining 2.0% of trait variance. The region harboring this SNP was captured by the RHM method as the most significant region associated with HDL, explaining 4.0% of trait variance and 8.7% of the total heritability. The meta-analysis further confirmed that ‘rs3764261' is the most significant SNP associated with HDL with a −log *P*-value of 379.14; this SNP explained 4.5% of the trait variance based on its reported allele frequency and estimated effect in the meta-analysis. The current study suggests that the RHM method performs better than the SSGWAS method in capturing the effective variants.

### RHM and SSGWAS vs meta-analysis

Table 3 presents comparisons between variance explained by meta-analysis, SSGWAS and RHM in the traits of interest. The meta-analysis identified 45 associated variants for HDL in addition to the two regions identified in this study. This is likely due in part to the sample size of the meta-analysis of >100 000 individuals compared with 2246 individuals in the current study. In addition, 34 out of 47 SNPs associated with HDL in the meta-analysis were not genotyped in the current study.

We estimated that the heritability explained by the 47 loci detected in the meta-analysis would be 12.2% for HDL using reported MAFs and effects in the meta-analysis data set. In addition, 13 of these SNPs genotyped in the current study would explain 6.4% of the total variance of HDL. Although just one of the 13 SNPs available out of the 47 was significant in the SSGWAS in the current study, these loci together explained 4.4% of total trait variance for HDL. Using the RHM method, regions harboring these 13 SNPs explained 8.5% of the total trait variance for HDL in our data. The results suggest that the RHM method has the potential to explain a larger proportion of variance in traits than the SSGWAS method in the regions for which meta-analysis hits are available in our data set. For associating SNPs reported by meta-analysis that are available in our data set, the MAF in meta-analysis and estimated allele frequency in the current study is plotted in Supplementary Figure 1. For the other 34 regions for which the meta-analysis hits are not available in our data set, the regional heritability estimated by RHM was calculated; the total heritability of HDL explained by all 47 regions by RHM was 19.4%.

For LDL, the most significant region detected by RHM was that harboring the SNP (‘rs4420638') that was most significantly associated in the meta-analysis of Teslovich *et al.* (2010). This SNP was estimated to explain 2.3% of the variance for LDL based on the meta-analysis results. In the RHM analysis, the region containing this SNP explained an estimated 4.8% of the trait variance. Neither this SNP nor any SNP in high linkage disequilibrium (*r*^2^ >0.90) with it was genotyped in the current study; therefore, the region was not detected by SSGWAS. This confirms the analysis of Nagamine *et al.* (2012b) that the RHM method has the power to detect the associated regions, even if the associated variant is not genotyped in the data.

The 37 loci detected in meta-analysis explain 12.1% of total trait variance for LDL. Only 6 of the 37 SNPs were genotyped in the current study, and these explained 0.8% of total variance in meta-analysis, 0.8% using the SSGWAS method and 0.6% using the RHM method.

No significantly associated SNP or region was detected in the current study for either TC or TG. Teslovich *et al.* (2010) reported 52 and 32 associated loci for TC and TG, respectively, with estimated heritabilities explained by these loci of 12.4 and 9.6%. From these meta-analysis associated variants, only 12 and 4 SNPs, for TC and TG, respectively, were available in the current study. Based on the meta-analysis results, these SNPs explained 1.4% and 0.5% of the total trait variances for TC and TG, respectively. The SSGWAS study also suggested similar percentage of variance for these SNPs with 1.2% and 0.4% for TC and TG, respectively. The RHM method showed a higher percentage of variance for the regions containing these genotyped SNPs of 2.6% and 4.9% for TC and TG, respectively. However, using the RHM method, the 52 regions in TC and the 32 regions in TG that harbored associated variants as reported by Teslovich *et al.* (2010) explained 10.4% and 6.4% of trait variance in the current study compared with the meta-analysis of 12.4% and 9.4% for TC and TG, respectively.

## Conclusion

Three significant regions detected by RHM provide evidence on the power of RHM under three different conditions. First, RHM can detect trait-associated regions that cannot be detected by the SSGWAS method by taking into account the nearby SNP effects. Second, this study suggests that when the SNP is not available in the data set, the RHM method can detect causative regions as this method uses effects of all SNPs in the region through the construction of a regional relationship matrix. Finally, the RHM method may be able to reduce the false discovery rate, a common problem with SSGWAS studies in populations that are characterized by a high inbreeding coefficient (Manenti *et al.*, 2009) specially for SNPs with high MAF (Tabangin *et al.*, 2009), by using nearby SNP information and the summation of SNP effects on a window.

## Data archiving

Summary statistics for SSGWAS and summary statistics for Regional Heritability Mapping are available from the Dryad Digital Repository: http://dx.doi.org/10.5061/dryad.6b65r. We have neither consent from individual participants nor appropriate ethical approval to permit full public release of data underlying this study. Researchers requiring access to the full data should contact Dr Caroline Hayward (caroline.hayward@igmm.ed.ac.uk).

## Figures and Tables

**Table 1:** Genome-wide significant RHM with 100-SNP windows overlapping and result from top 10-SNP window in each region

*Trait*	*Chr*	*100-SNP*						*10-SNP*					
		*Window no.*	*SNP and position (bp)*		*LRT*	−*Log* P-*value*	*RH*	*Window no.*	*SNP and position (bp)*		*LRT*	−*Log* P-*value*	*RH*
			*Start*	*End*					*Start*	*End*			
HDL	16	81	rs2291956 55 249 719	rs496772 56 069 867	26.42	7.15	4.0%	807	rs247615 55 542 264	rs289735 55 587 063	54.26	13.35	4.0%
	15	66	rs4646583 56 093 843	rs7174386 56 716 605	24.02	6.61	2.7%	663	rs1373656 56 457 727	rs6494006 56 517 863	25.86	7.02	3.5%
LDL	19	71	rs203713 49 860 337	rs8111071 50 999 246	23.20	6.40	4.8%	706	rs2075650 50 087 459	rs3786507 50 240 095	47.76	11.91	7.3%

Abbreviations: Chr, chromosome; HDL, high-density lipoprotein; LDL, low-density lipoprotein; LRT, likelihood ratio test; RH, regional heritability; RHM, regional heritability mapping; SNP, single-nucleotide polymorphism.

**Table 2:** Explained variance by SNP/region using meta-analysis, SSGWAS and RHM methods for captured SNPs

*Chromosome*	*SNP*	*Trait*	*Meta-analysis*				*SSGWAS*	*RHM*	
			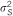	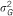	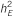	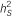	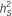	h^2^*_win_*10	h^2^*_win_*100
16	rs3764261	HDL	5.00	13.64	12.2%	4.5%	2.0%	4.0%	4.0%
15	rs1532085	HDL	1.00	13.64	12.2%	0.9%	1.2%	3.5%	2.7%
10	rs3758562	HDL	NA	13.64	12.2%	NA	1.3%	1.1%^NS^	1.3%^NS^
19	rs4420638	LDL	14.38	75.68	12.1%	2.3%	NA	7.3%	4.8%

Abbreviations: HDL, high-density lipoprotein; LDL, low-density lipoprotein; LRT, likelihood ratio test; NA, not available; NS, not significant; RHM, regional heritability mapping; SNP, single-nucleotide polymorphism; SSGWAS, single SNP genome-wide association study.

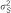
: explained variance by the SNP; 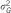: total genetic variance; 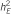: explained trait heritability by all meta-analysis hits; 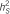: explained heritability by the SNP; 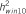: heritability of the region using 10-SNP window by RHM method; 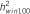: heritability of the region using 100-SNP window by RHM method.

**Table 3:** Variance comparison between meta-analysis, SSGWAS and RHM

*Method*	*HDL*		*LDL*		*TC*		*TG*	
	*N*_*S*_	*PVE*	*N*_*S*_	*PVE*	*N*_*S*_	*PVE*	*N*_*S*_	*PVE*
*SSGWAS*								
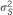	13	4.4%	6	0.8%	12	1.2%	4	0.4%
								
*RHM*								
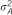 a	47	19.4%	37	12.4%	52	10.4%	32	6.4%
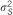 a	13	8.5%	6	0.6%	12	2.6%	4	4.9%
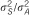	13/47	43.8%	6/37	2.3%	12/52	25.0%	4/32	76.5%
								
*Meta-analysis*								
*σ*_*A*_^2^	47	12.2%	37	12.1%	52	12.4%	32	9.6%
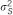	13	6.4%	6	0.8%	12	1.4%	4	0.5%
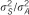	13/47	52.6%	6/37	6.7%	12/52	11.5%	4/32	5.2%

Abbreviations: HDL, high-density lipoprotein; LDL, low-density lipoprotein; NS, number of SNPs; PVE, proportion of trait variance explained; RHM, regional heritability mapping; SNP, single-nucleotide polymorphism; SSGWAS, single SNP genome-wide association study; TC, total cholesterol; TG, triglycerides.

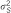
: explained variance by associating SNP reported by meta-analysis that are available in our data set; 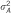: explained variance of all associating SNPs reported by meta-analysis.

a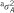 and 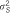 for RHM are explained variances by the regions containing the SNPs.
