# Supplementary material for: Regional heritability mapping method helps explain missing heritability of blood lipid traits in isolated populations
Source: Heredity (Edinb). 2015 Dec 23;116(3):333–8. doi: 10.1038/hdy.2015.107 (PMC4751621; doi:10.1038/hdy.2015.107)

Supplementary

Supplementary Table 1. Mean and standard deviation of the distributions of blood lipid trait concentrations in plasma in three isolated Southern European populations after removing outliers (mmol/L)

| Population Name | Sample size | HDL | |  | LDL | |  | TC | |  | TG | |
| --- | --- | --- | --- | --- | --- | --- | --- | --- | --- | --- | --- | --- |
|  |  | Mean | SD |  | Mean | SD |  | Mean | SD |  | Mean | SD |
| Vis | 923 | 1.602 | 0.353 |  | 3.795 | 1.059 |  | 6.046 | 1.144 |  | 1.622 | 0.778 |
| Korcula | 856 | 1.458 | 0.330 |  | 3.798 | 0.997 |  | 5.880 | 1.148 |  | 1.365 | 0.622 |
| Split | 467 | 1.394 | 0.325 |  | 3.818 | 1.035 |  | 5.832 | 1.190 |  | 1.356 | 0.674 |

Supplementary Table 2. Single SNP genome-wide association study (SSGWAS) results SNPs with –log P value more than 5.00 in each trait of interest

| Trait | SNP | Chromosome | Position | Allele B | Allele B  Effect | MAF |  | -Log P |
| --- | --- | --- | --- | --- | --- | --- | --- | --- |
| HDL | rs7499892 | 16 | 55,564,091 | A | -0.270 | 0.164 |  | 10.38 |
|  | rs3764261 | 16 | 55,550,825 | A | 0.214 | 0.312 |  | 10.15 |
|  | rs3758562 | 10 | 72,032,086 | G | -0.172 | 0.330 |  | 6.84 |
|  | rs1532085 | 15 | 56,470,658 | A | 0.171 | 0.320 |  | 6.70 |
|  | rs1532624 | 16 | 55,562,980 | A | 0.160 | 0.408 |  | 6.55 |
|  | rs16940027 | 15 | 56,381,022 | G | 0.286 | 0.067 |  | 5.57 |
| LDL | rs17111051 | 5 | 149,574,450 | A | 0.344 | 0.044 |  | 5.54 |
|  | rs13292550 | 9 | 17,268,726 | G | -0.155 | 0.283 |  | 5.26 |
|  | rs3014994 | 12 | 66,636,447 | G | 0.140 | 0.485 |  | 5.22 |
|  | rs2032953 | 2 | 231,744,037 | A | -0.143 | 0.326 |  | 5.05 |
| TC | rs1733743 | 10 | 149,574,450 | A | 0.154 | 0.301 |  | 5.33 |
| TG | rs12633980 | 3 | 126,789 | C | 0.192 | 0.158 |  | 5.39 |
|  | rs9837120 | 3 | 194,953,909 | G | -0.147 | 0.345 |  | 5.22 |
|  | rs473224 | 15 | 56,524,633 | A | 0.204 | 0.129 |  | 5.14 |

Supplementary Table 3. Regional heritability mapping method (RHM) results for 100-SNPs windows with -logP more than 3.40 in all traits

| Trait | Chr | Window | position (bp) | | LRT | -logP | RH |
| --- | --- | --- | --- | --- | --- | --- | --- |
|  |  |  | Start | End |  |  |  |
| HDL | 16 | 81 | 55,249,719 | 56,069,867 | 26.42 | 7.15 | 4.0% |
|  | 15 | 66 | 56,093,843 | 56,716,605 | 24.02 | 6.61 | 2.7% |
|  | 16 | 80 | 54,703,868 | 55,634,477 | 23.36 | 6.46 | 4.0% |
|  | 15 | 65 | 55,705,113 | 56,399,978 | 13.88 | 4.29 | 1.7% |
|  | 15 | 67 | 56,402,226 | 57,386,341 | 13.08 | 4.10 | 3.0% |
| LDL | 19 | 71 | 49,860,337 | 50,999,246 | 23.2 | 6.42 | 4.8% |
|  | 19 | 70 | 49,455,904 | 50,471,475 | 23.1 | 6.40 | 6.2% |
| TC | 8 | 303 | 140,258,145 | 140,831,665 | 13.72 | 3.97 | 2.5% |
|  | 8 | 302 | 139,955,962 | 140,577,261 | 13.70 | 3.97 | 2.5% |
|  | 12 | 107 | 52,001,135 | 53,208,607 | 13.14 | 3.84 | 1.8% |
| TG | 20 | 46 | 15,542,367 | 16,131,412 | 12.34 | 3.65 | 1.8% |
|  | 3 | 369 | 194,959,757 | 195,653,793 | 11.80 | 3.53 | 1.8% |

Chr: Chromosome; RH: Regional Heritability;

Supplementary Figure 1: The minor allele frequency reported in the meta-analysis study for available SNPs in our data set and estimated allele frequency for them in the current study. The black line presents one to one ratio.


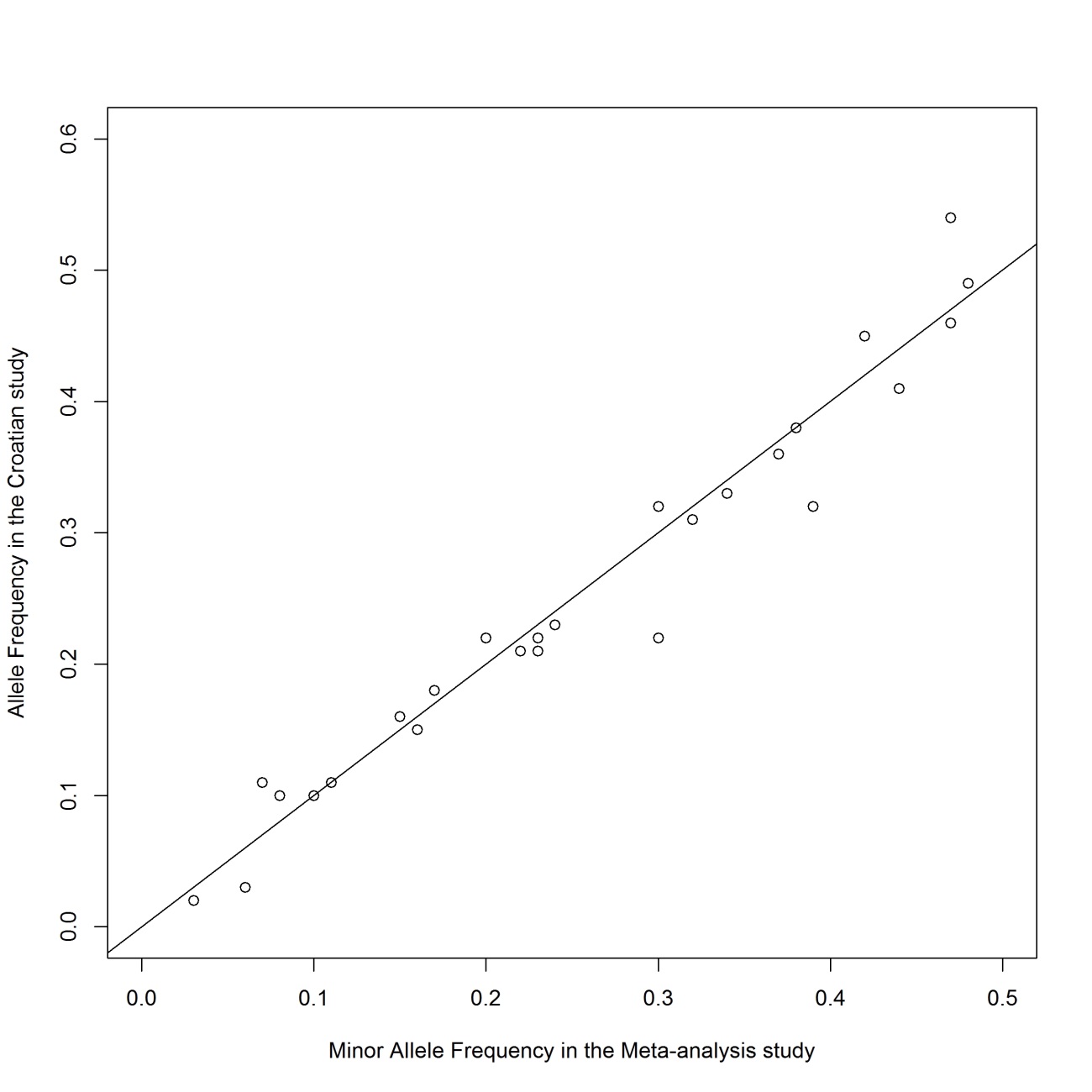

Supplement: Supplementary Information [file hdy2015107x1.docx]
